# Influence of Silica Modulus and Curing Temperature on the Strength of Alkali-Activated Volcanic Ash and Limestone Powder Mortar

**DOI:** 10.3390/ma14185204

**Published:** 2021-09-10

**Authors:** Adeshina Adewale Adewumi, Mohd Azreen Mohd Ariffin, Mohammed Maslehuddin, Moruf Olalekan Yusuf, Mohammad Ismail, Khaled A. Alawi Al-Sodani

**Affiliations:** 1 Department of Civil Engineering, University of Hafr Al Batin, Hafar Al-Batin 31991, Saudi Arabia; walasco2010@gmail.com (A.A.A.); moruf@uhb.edu.sa (M.O.Y.); kalsodani@uhb.edu.sa (K.A.A.A.-S.); 2 School of Civil Engineering, Faculty of Engineering, Universiti Teknologi Malaysia (UTM), Johor Bahru 81310, Malaysia; mohammad@utm.my; 3 Forensic Engineering Centre, Institute for Smart Infrastructure & Innovation Construction, School of Civil Engineering, Faculty of Engineering, Universiti Teknologi Malaysia (UTM), Johor Bahru 81310, Malaysia; 4 Integrated Center for Research on Construction and Building Materials, King Fahd University of Petroleum and Minerals, Dhahran 31261, Saudi Arabia; muddin@kfupm.edu.sa

**Keywords:** alkali-activated mortar, volcanic ash, limestone powder, microstructure, aluminosilicate, silica modulus, curing temperature

## Abstract

This present study evaluates the effect of silica modulus (M_s_) and curing temperature on strengths and the microstructures of binary blended alkali-activated volcanic ash and limestone powder mortar. Mortar samples were prepared using mass ratio of combined Na_2_SiO_3(aq)_/10 M NaOH_(aq)_ of 0.5 to 1.5 at an interval of 0.25, corresponding to M_s_ of 0.52, 0.72, 0.89, 1.05 and 1.18, respectively, and sole 10 M NaOH_(aq)_. Samples were then subjected to ambient room temperature, and the oven-cured temperature was maintained from 45 to 90 °C at an interval of 15 °C for 24 h. The maximum achievable 28-day strength was 27 MPa at M_s_ value of 0.89 cured at 75 °C. Samples synthesised with the sole 10 M NaOH_(aq)_ activator resulted in a binder with a low 28-day compressive strength (15 MPa) compared to combined usage of Na_2_SiO_3(aq)_/10 M NaOH_(aq)_ activators. Results further revealed that curing at low temperatures (25 °C to 45 °C) does not favour strength development, whereas higher curing temperature positively enhanced strength development. More than 70% of the 28-day compressive strength could be achieved within 12 h of curing with the usage of combined Na_2_SiO_3(aq)_/10 M NaOH_(aq)_. XRD, FTIR and SEM + EDX characterisations revealed that activation with combined Na_2_SiO_3(aq)_/10 M NaOH_(aq)_ leads to the formation of anorthite (CaAl_2_Si_2_O_8_), gehlenite (CaO.Al_2_O_3_.SiO_2_) and albite (NaAlSi_3_O_8_) that improve the amorphosity, homogeneity and microstructural density of the binder compared to that of samples synthesised with sole 10 M NaOH_(aq)_.

## 1. Introduction

Concrete is the bedrock of our built environment. The construction of critical infrastructures for social and economic development consumed approximately 35 billion tons of concrete inclusive of steel, wood and aluminium [1]. The choice of concrete as the most widely used construction material was attributed to its favourable mechanical and durability performance couple with its global availability and relatively low cost [2]. Cement is an essential binding material in concrete. Cement global production was estimated at approximately 4.1 billion tons in 2017, with an expected four-fold increase by 2050 [3]. Despite the numerous advantages of cement, it significantly contributes to 5–8% of global CO_2_ resulting in climate change [4,5]. To mitigate the proliferation of CO_2_ into the environment, researchers are focusing on developing viable and sustainable alternative friendly novel binders derived from industrial or natural waste materials that will ensure an 80% CO_2_ reduction level by 2050 in conduction with a range of CO_2_ capturing technologies [3].

Alkali activated material (AAM) is an inorganic binder developed by the reaction of an alkali metal source with solid aluminosilicate-based materials [6]. AAM was identified as a promising alternative binder to OPC due to its superior mechanical, durability and thermal properties [7]. The use of AAM was first patented by Hans Kuhl in 1908 [8], the reaction between vitreous slag and an alkali sulphate in the presence or absence of alkaline earth oxides or hydroxides was studied. It was found that the developed binder was of equal performance with OPC. However, the science behind this discovery was not understood until 1940. The first scientific explanation for this binder development was given by Purdon in 1940 [9]. Purdon (1940) developed a binder by using slag as a base material and NaOH_(aq)_ as an activator. The outcome of the findings showed better performance in terms of strength and durability. According to Purdon (1940), alkali activation using NaOH_(aq)_ involved two stages. First was the dissolution of Si and Al from base materials. The second was the regeneration of alkali and the formation of aluminosilicate hydrate (A-S-H).

AAM is classified into high and low calcium systems; the main product of the high calcium system, such as alkali-activated blast furnace slag, is C-A-S-H gel, while the resultant product of low calcium system, such as Class F fly ash and metakaolin, is N-A-S-H gels [10]. The two systems have good mechanical and durability performances. However, some drawbacks are associated with the individual usage of the system; for instance, fast setting and high shrinkage are inherent in a high calcium system [11,12], while long setting times and continuous high curing temperatures characterised low calcium system [13,14]. These drawbacks have limited their practical applications. To overcome the drawbacks inherent in the individual system, the synergy between the two systems resulted in an improved fresh and hardened properties. Several studies investigated the blended alkaline matrices that involve precursors highly rich in calcium and aluminosilicate compounds. Results show improved setting times, workability, strength and durability performance [6,12,15]. The reaction products of the blended alkaline systems are cross-linked C-A-S-H, N-A-S-H and (N, C)-A-S-H [7].

Volcanic ash (VA), a type of natural pozzolan, is a suitable base material for geopolymer binder due to its high silica content and are abundantly available in countries with a volcanic record such as Iran, Saudi Arabia, Turkey, Cameroon, Japan, Jordan, Kuwait, Colombia and the USA [16]. The reactivity of the VA geopolymer can be enhanced by using a high concentration of an alkaline solution, high curing temperature and binary blending with secondary cementitious material [17]. Moreover, due to the very low content of CaO in VA, delay in setting was reported to be more than seven days, which makes the demolding of samples difficult [17]. To enhance the workability and the mechanical strength, binary blending between silica and CaO containing precursors such as slag and limestone were reported to be effective [18,19]. About 21.2 million tons of LSP are produced in the United Kingdom, while in Greece, 18 million tons are generated, and Turkey generates 30 million tons annually [20]. Dust from LSP leads to environmental and health hazards such as cancer and asthma [21]. Apart from the environmental benefits of using limestone powder in OPC concrete system, limestone powder was shown to have a filler effect on OPC concrete system, provide nucleation site which increases the hydration of C_3_S, partially involved in the formation of C-S-H gels, thereby enhancing the workability and strength of OPC concrete system [22,23,24]. The impact of calcite on alkali-activated metakaolin by Yip et al. [25] showed an improvement in the strength development at calcite substitution of less than 20%, and it was revealed that the gained in strength was mainly due to the surface binding effect of calcite while the charge balancing effect of Ca^2+^ released from calcite was very low. 

AAM performance depends on the types of alkaline activators, curing methods, curing temperature and duration [18,26,27]. However, silica modulus and curing temperatures influence the reaction kinetics of activated binders significantly. The effect of silica modulus on a binary mix of natural pozzolan (NP) and slag alkali-activated binder (AAB) was studied [28]. Ibrahim et al. investigated the effect of silica modulus on alkali-activated natural pozzolan concrete [29]. Curing temperature is another very important parameter that affects the strength development of alkali-activated binders. The authors of [30] investigated the effects of temperature on the strength development of alkali-activated fly ash-slag binder. It was found that curing at low temperatures hindered quick strength development of fly-ash-based alkali-activated binder due to low reactivity and dissolution of fly ash at a lower temperature of 25 °C. However, at a higher curing temperature of 60 °C and 90 °C significant strength was reported. At higher temperatures, the rate of reaction is faster, and about 70% gain in strength was reported by Kong et al. [31]. The most beneficial curing temperature of 120° was reported for fly-ash-based geopolymer mortar [32]. Despite numerous works on the effects of alkali activators on the alkali-activated binder, there is a limited study concerning the role of silica modulus and curing temperature on mechanical, and microstructure properties of alkaline binary blended volcanic ash powder and limestone powder mortar. 

This paper reports the findings of the influence of silica modulus and curing temperature on the strength and microstructure of alkali-activated LSP/VA mortar. It is expected that the outcomes of this study will help in understanding the impact of alkaline activators and curing temperature and also contribute to a better understanding of the impact of silica modulus and curing temperature on the characteristics of the alkali-activated volcanic ash/limestone powder mortar. The utilisation of VA/LSP will be an addition to sustainable alternative binders and waste reduction.

## 2. Materials and Methods

### 2.1. Based Materials Characterisation

#### 2.1.1. Volcanic Ash Powder and Limestone Powder 

The volcanic ash was provided by Imerys minerals Arabia, Rabigh, Kingdom of Saudi Arabia, and the limestone powder (LSP) was obtained from the quarry as a waste. The LSP was oven-dried at 105 °C ± 5 °C for 24 h. The chemical compositions of volcanic ash and the LSP determined using an X-ray fluorescence (XRF) spectrometer are provided in Table 1. It was found that LSP is composed mainly of CaO (94.1%) with a very low content of silica (2.5%) and alumina (0.8%); conversely, VA has silica (74%) as its main component with moderate content of alumina (13%). This was the reason for the binary blended alkaline activated binder using these two materials. 

#### 2.1.2. Synthesis of Alkaline Activator

Industrial purchased aqueous sodium silicate (SS) and sodium hydroxide (NH) were used as alkaline activators. The initial silica modulus (M_s_ = SiO_2_/Na_2_O) of SS was 3.3, and the percentage composition of the Na_2_SiO_3(aq)_ is as follows; H_2_O: 62.11%, SiO_2_: 29.13% and Na_2_O: 8.76%. A 10 M NaOH_(aq)_ solution was prepared by dissolving 404.4 g of NaOH pellet (99% assays) into 1 L of distilled water and was kept at ambient temperature for at least 24 h for cooling before being used. The SS and NS were combined at a ratio of x ($\frac{SS}{NH})$, where x varied from 0.5 to 1.5 at an interval of 0.25.

#### 2.1.3. Aggregates

Dune desert sand passing the gradation size requirement of ASTM C33 was used as the fine aggregate (FA) [33]. The fineness modulus of sand was 1.82, with 2.63 as the specific gravity.

### 2.2. Experimental Program

#### 2.2.1. Mix Design

The mortar was prepared using a binary combination of 40% VA and 60% LSP [19]. The samples were prepared using a mass ratio of Na_2_SiO_3(aq)_/10 M NaOH_(aq)_ (SS/NH) of 0.5 to 1.5 at an interval of 0.25. The samples were designated as AANLM_x_ (alkali-activated VA/LSP mortar), where x = $\frac{SS}{NH}$. A total of six AANLM_x_ (where x = 0, 0.5, 0.75, 1, 1.25 and 1.5) mixtures were prepared to study the combined effect of SS and NH (silica modulus), the sole effect of 10 M NaOH_(aq),_ and the effect of curing temperature on strengths and microstructures of the developed mortar. All the mixtures were prepared using a constant fine aggregate (FA) to the binder ratio of two based on the beneficial value from the preliminary trial mix. The alkaline activator to binder ratio of 0.5 was used for the mortar and 0.25 for the paste. The free water to precursor (pozzolanic material) ratio of 0.1 was used in all the mixtures based upon the preliminary trial mix to attain a workable mixture. Table 2 shows the proportion of the constituent materials in the alkali-activated mortar mixtures.

#### 2.2.2. Mortar Mixing, Placing and Curing

The mixing procedure adopted in this work is the same as reported by previous researchers [19,34,35,36,37].

The required quantities of constituent materials were measured and mixed in batches in the 5.0 L capacity Hobart planetary bench mixer. The mixing of the materials was performed in two stages. In the initial stage, the VA and LSP powder and sand were mixed in a dry condition for 3 min. In the last stage, the alkaline solution (NaOH_(aq)_ + Na_2_SiO_3(aq)_) and water were added for the wet mixing stage, which involves low speed mixing for 2 min and another 4 min for fast or higher speed mixing until a homogeneous mixture was achieved, the total mixing time was about 9 to 10 min to ensure the homogeneity of the mix. Thereafter, the mortar was placed in the oil-smeared steel moulds of 50 × 50 × 50 mm^3^ in two layers, and each layer was vibrated on the vibrating table for 30 s to remove any entrapped air from the mixture. Then, the surface was carefully smoothened with a trowel to have a smooth finish. After the placement, consolidation and finishing of the mortar, the specimens were covered with a plastic sheet to prevent moisture loss and kept in the laboratory at 20 ± 5 °C for 24 h. After 24 h of casting, the cubes were de-moulded and placed in zip plastic bags to avoid evaporation of moisture. The samples were then subjected to temperature curing in an oven maintained at room temperature (20 ± 5 °C) and various curing temperatures of 45, 60, 75 and 90 °C for 24 h. After that, the specimens were cured under a normal room temperature condition of 20 ± 5 °C until the age of testing (1, 3, 7, 14 and 28 days). The compressive strength of the mortar was determined in accordance with ASTM C 150 [38] on the 50 × 50 × 50 mm^3^ cube specimens using a Matest digital compression testing machine. The compressive strength of the specimens was determined after 1, 3, 7, 14 and 28 days of curing. Three specimens were tested at each age, and the average compressive strength value was recorded.

## 3. Result and Discussion

### 3.1. Physical and Mineralogy Characteristics of Base Materials

**Figure 1:**
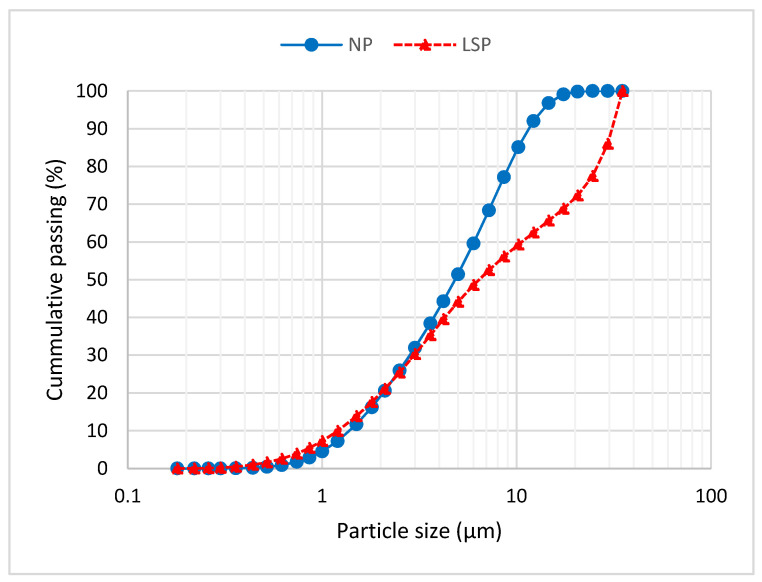
Particle size distribution curve of LSP and VA.

The physical properties such as specific gravity, mean particle size and specific surface area of VA and LSP are depicted in Table 3. VA has a smaller mean particle size of 5.77 µm, while LSP has a larger mean particle size of 12.05 µm. The specific surface area of VA is larger than that of LSP by about five times. The particle size distribution curves of LSP and VA are provided in Figure 1.

**Figure 2:**
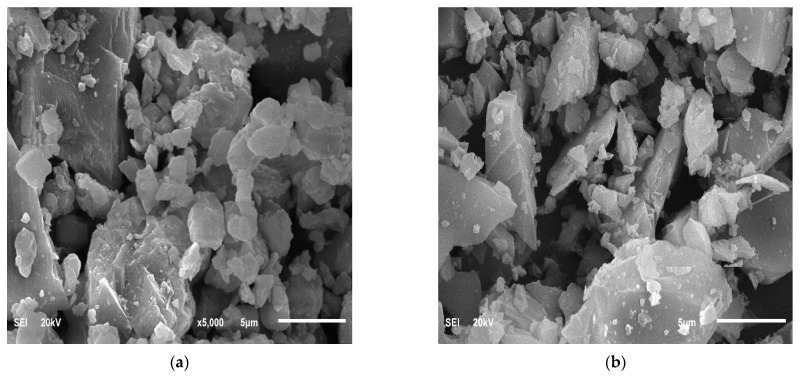
SEM images of (**a**) Raw limestone powder (**b**) Raw volcanic powder.

**Figure 3:**
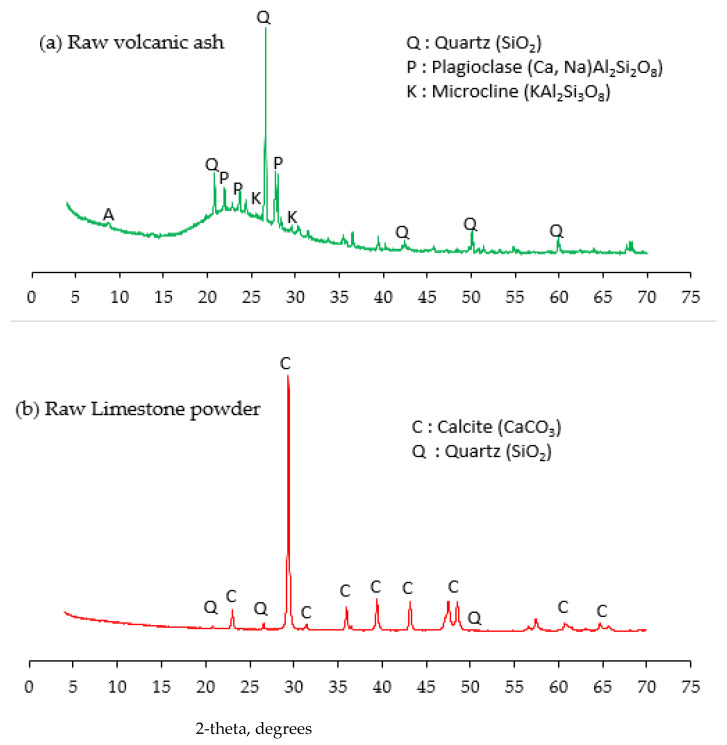
XRD pattern: (**a**) Raw volcanic ash (**b**) Raw limestone powder.

Figure 2 reveals a micrograph of LSP and VA examined using a JSM-5800LV scanning electron microscope (SEM). LSP has a large size with a round edge polycrystal-like shape, while VA has a smaller particle size with an angular shape coupled with elongated flakiness. Figure 3 shows the XRD result for the LSP and VA. LSP is crystalline in nature and contains mainly calcite (CaCO_3_), while VA is amorphous in nature and contains plagioclase (Ca,Na)Al_2_Si_2_O_8_, quartz (SiO_2_) and microcline (KAl_2_Si_2_O_8_).

### 3.2. Effect of Silica Modulus on Workability of AANL

**Figure 4:**
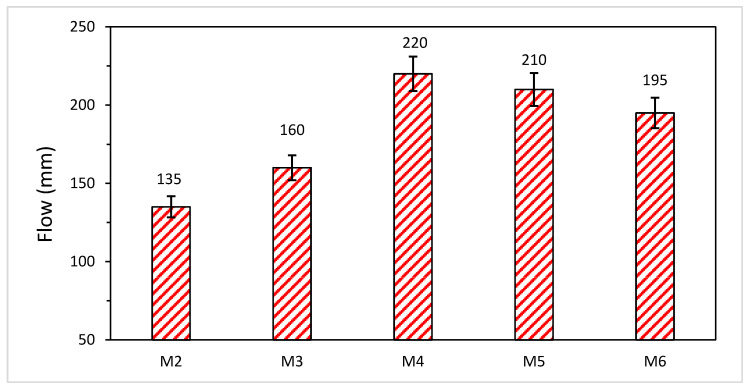
Flowability of the AANLM at varying Na_2_SiO_3(aq)_/10 M NaOH_(aq)_ (SS/NH) ratio. (M2 = AANLM_0.5_, M3 = AANLM_0.75_, M4 = AANLM_1_, M5 = AANLM_1.25_, M6 = AANLM_1.5_).

The workability of AANLM_x_, where x is the mass ratio of Na_2_SiO_3(aq)_/10 M NaOH_(aq)_ (x = $\frac{SS}{NH}$, x = 0, 0.5, 0.75, 1, 1.25 and 1.5), is presented in Figure 4. The mixtures were prepared to study the combined effect of SS and NH i.e., (x = 0.5, 0.75, 1, 1.25 and 1.5) and the sole effect of 10 M NaOH_(aq)_ (x = 0). The silica modulus (SiO_2_/Na_2_O) was calculated from the mass ratios as tabulated in Table 4. The corresponding silica modulus (M_s_) of x = 0, 0.5, 0.75, 1, 1.25 and 1.5 are 0, 0.52, 0.72, 0.89, 1.05 and 1.18, respectively.

**Figure 5:**
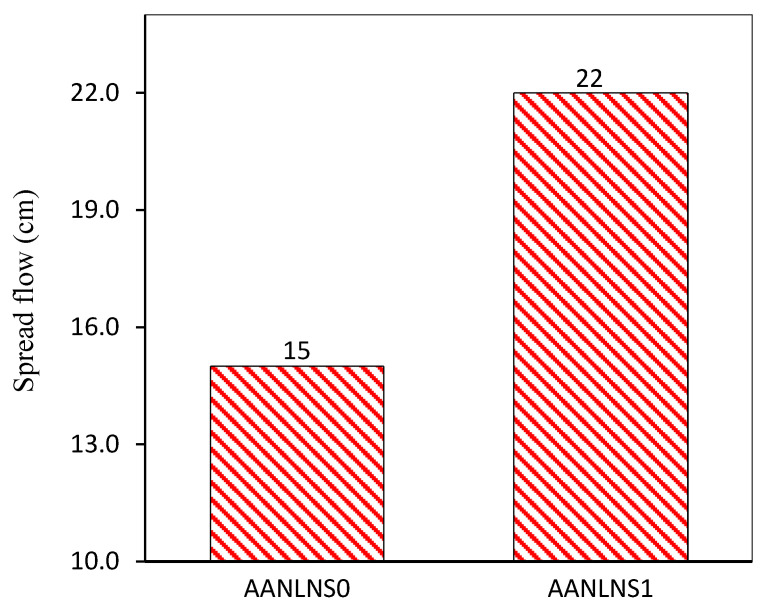
Flowability of the AANLM activated using NaOH only and the combination of NaOH_(aq)_/Na_2_SiO_3(aq)_.

The workability of the AANLM increases as the $\frac{SS}{NH}$ increased from 0.5 to 1 until optimum values of silica modulus of 0.89, and thereafter, a reduction in the workability was observed. There were 18.51% and 62.96% increments in AANLM_0.75_ and AANL_1_, respectively, in comparison with AANLM_0.5_, as shown in Figure 4. However, a slight reduction was observed when the silica modulus increased from 0.89 to 1.05 and 1.18. There was a 4.55% and 11.36% reduction in AANLM_1.25_ and AANLM_1.5_ in comparison to AANLM_1_. It is what noticing that the combination of Na_2_SiO_3_ and NaOH_(aq)_ resulted in higher workability than using only NaOH_(aq)_, as shown in Figure 5. The higher workability observed in AANLM_1.0_ could be as a result of higher H_2_O/SiO_2_ (5.46) H_2_O/Na_2_O (6.12) present in the mix compared to the AANLM_0_.

### 3.3. Effect of Silica Modulus on Compressive Strength Development

The initial oxide composition and the oxide composition ratio of the alkaline activators are shown in Table 4 [39]. NaOH_(aq)_ and Na_2_SiO_3(aq)_ dissolution processes are shown in Equations (1)–(4).

(1) $$x{\mathrm{NaOH}}_{\left(\mathrm{aq}\right)}+yH_{2}O_{\left(l\right)}\to \frac{x}{2}{\mathrm{Na}}_{2}O+\frac{x}{2}H_{2}O+yH_{2}O_{\left(l\right)}$$

Na_2_SiO_3(aq)_ → Na_2_O + SiO_2_ + H_2_O (2)

(Na_2_O: 8.76%, SiO_2_: 29.13%, H_2_O: 62.11%) (3)

**Figure 6:**
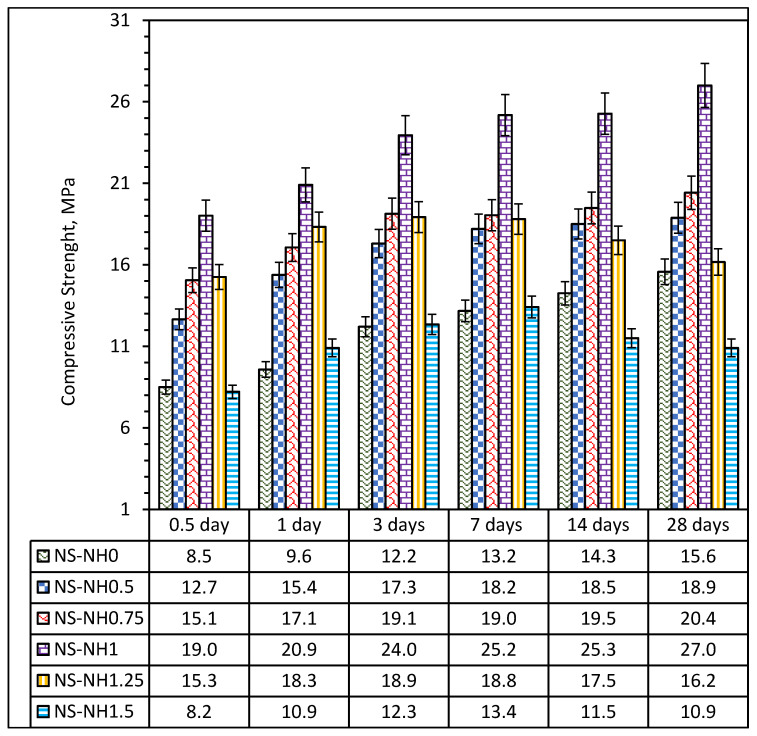
Compressive strength of AANL mixes with varying silica modulus.

*z* H_2_O_(l)_ → *z* H^+^ + *z* OH^−^ (4)

where *x*, *y* and *z* represent the molar concentration of NaOH_(aq)_, added distilled water and the mixing water, respectively. The total mass concentrations of Na_2_O, SiO_2_ and H_2_O and the mass ratio of silica modulus, (M_s_) SiO_2_/Na_2_O, H_2_O/Na_2_O and H_2_O/SiO_2_ are as shown in Table 4 increase in Na_2_SiO_3(aq)_/10 M NaOH_(aq)_ (x = $\frac{SS}{NH}$) from 0 to 1.5 leads to an increase in the total SiO_2_ and a decrease in the total Na_2_O and the H_2_O present in the mix proportion. Furthermore, the silica modulus (SiO_2_/Na_2_O) and H_2_O/SiO_2_ increases while the H_2_O/Na_2_O decrease as $\frac{SS}{NH}$ increased, as shown in Table 4. The impact of variation in the silica modulus on the compressive strength of the synthesised alkali-activated mortar is shown in Figure 6. Generally, the early compressive strength for all the mixes increases with the curing age till optimum values of silica modulus (0.89), and thereafter, a reduction in the compressive strength was observed. It should be noted that, after the 7-day strength, the mixes after the optimum silica modulus, i.e., silica modulus 1.25 and 1.18, showed a drop in strength.

The maximum strength of 27 MPa was obtained in AANLM_1_ with silica modulus of 0.89 after 28 days of curing; however, the compressive strength reduced sharply by 40% and 62%, respectively, in AANLM_1.25_ and AANLM_1.5_ due to the presence of more SiO_2_ than Na_2_O in the mix, as shown in Table 4. At lower silica modulus (0–0.72) below the optimum silica modulus (0.89), the compressive strength recorded was lower due to the presence of excess alkalis (Na_2_O) in the mixture, which caused negative effects such as efflorescence and brittleness of the binder product. Above the optimum silica modulus (0.89), the compressive strength recorded decreased drastically due to the presence of excess alkalis (SiO_2_) in the mixture. The excess SiO_2_ caused a reduction in pH, increase in viscosity and degree of polymerisation of silicate species of alkaline solution leading to a reduction in the reactivity of alkaline solution [17]. The trends observed clearly show the dependency of compressive strength development on the silica modulus of the alkaline activators. Silica modulus above and below the optimal values was found to be not suitable for achieving higher compressive strength. Similar trends were reported previously in the literature [17].

### 3.4. XRD Characterisation of Varied Silica Modulus on the Mortar Binder

**Figure 7:**
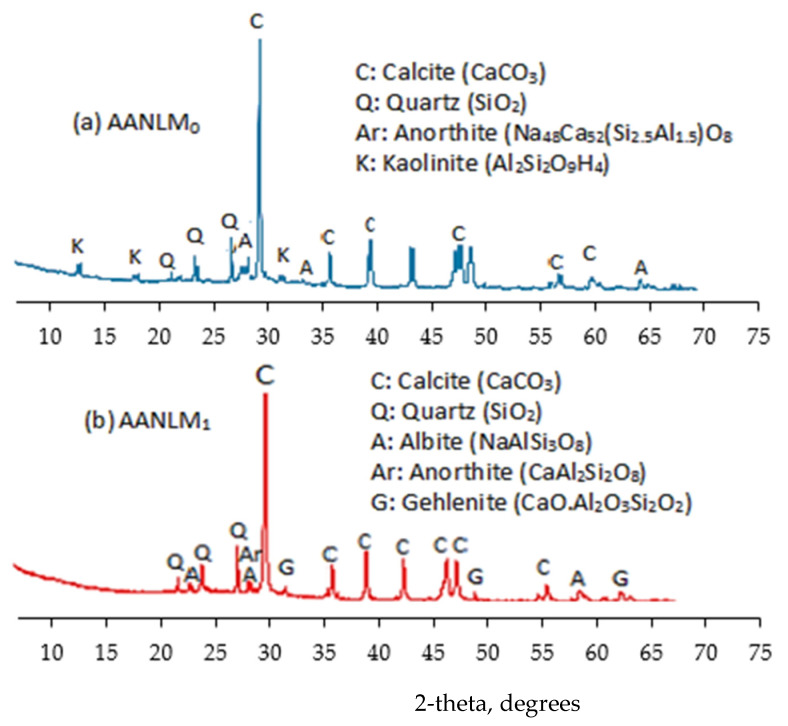
X-ray diffractograms of binder using (**a**) M_s_ = 0 and (**b**) M_s_ = 0.89.

The XRD spectra of the binder product that gave the best and worst results for compressive strength are shown in Figure 7. Sole usage of sodium hydroxide solution (10 M NaOH_(aq)_) as alkaline activator resulted in the formation of calcite (CaCO_3_), quartz (SiO_2_), anorthite (Na_48_Ca_52_(Si_2.5_Al_1.5_)O_8_ and kaolinite (Al_2_Si_2_O_9_H_4_). However, when a combined sodium hydroxide solution and sodium silicate were used, the alkaline activated products formed were anorthite (CaAl_2_Si_2_O_8_), gehlenite (CaO.Al_2_O_3_.SiO_2_) and albite (NaAlSi_3_O_8_). Anorthite (CaAl_2_Si_2_O_8_) and gehlenite (CaO.Al_2_O_3_.SiO_2_) compounds are similar to the calcium-aluminosilicate-hydrate (C-A-S-H) compound. The use of combined sodium hydroxide solution and sodium silicate enhanced the strength development than using only sodium hydroxide solution.

### 3.5. FTIR Analysis of Silica Modulus Effect

**Figure 8:**
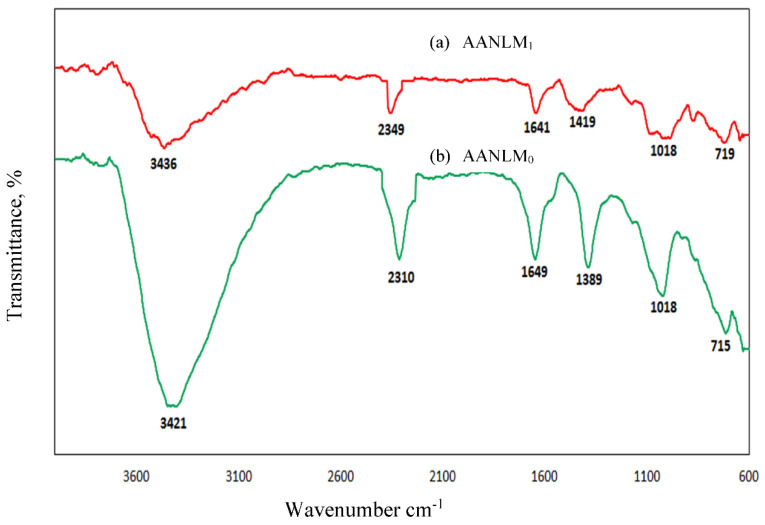
FTIR spectra of binder (**a**) activated using (M_s_ = 0) and (**b**) (M_s_ = 0.89).

Figure 8 shows the FTIR spectra of AANLM binder, activated using only 10M NaOH_(aq)_ (M_s_ = 0) and combined NaOH_(aq)_ and Na2SiO3_(aq)_ (M_s_ = 0.89). From Figure 8a,b, The O–H stretching is observed at broad bands located at 3700–3000 cm^−1^ and H–O–H bending at broad bands located at 2400–2300 cm^−1^ and 1700 cm^−1^ to 1600 cm^−1^ were observed in both mix matrix. The O–H and H–O–H bonds observed in the binder were associated with the weakly bound water molecules present on the surface or in the cavities of the binder [35,40]. The binder activated with only 10 M NaOH_(aq)_ (i.e., M_s_ = 0) has a broader trough compared to the one activated with M_s_ of 0.89; this indicated the presence of more weak O–H and H–O–H bonds in the former than the latter. This could cause the low strength observed in the sample synthesised with M_s_ = 0.

The presence of stretching vibration of CO_3_^2−^ observed at a wavenumber of 1419 cm^−1^ in AANLM_1_ and at a wavenumber of 1389 cm^−1^ in AANLM_0_ indicate the occurrence of carbonation in both binders, which could contribute to the densification of the binder, thereby enhancing their strength development. Intensity band in the FTIR spectrum of both binders at 1018 cm^−1^ associated with the Si–O–T (T = Si or Al) asymmetric vibration was broader and stronger in AANLM_1_ than AANLM_0_ (Figure 8b). Furthermore, the presence of in-plane bending vibration of C–O was observed at a band of the weak and broad absorption peak of 715 cm^−1^ for both samples.

### 3.6. SEM + EDX Characterisation of Silica Modulus Effect

**Figure 9:**
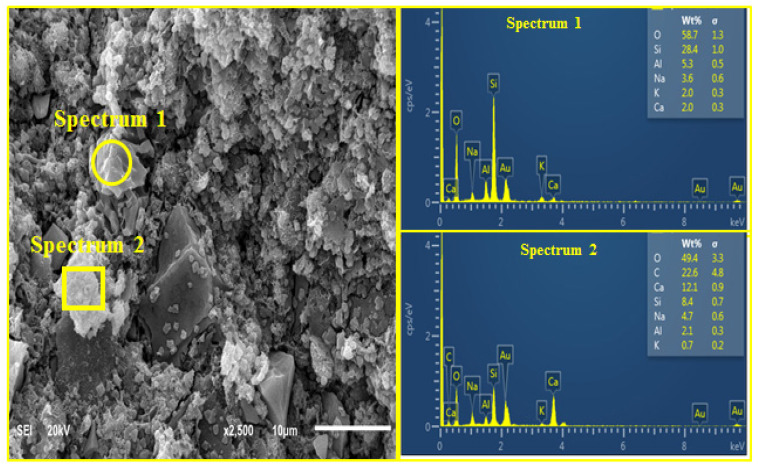
SEM + EDX result of AANL activated using only 10 M NaOH.

**Figure 10:**
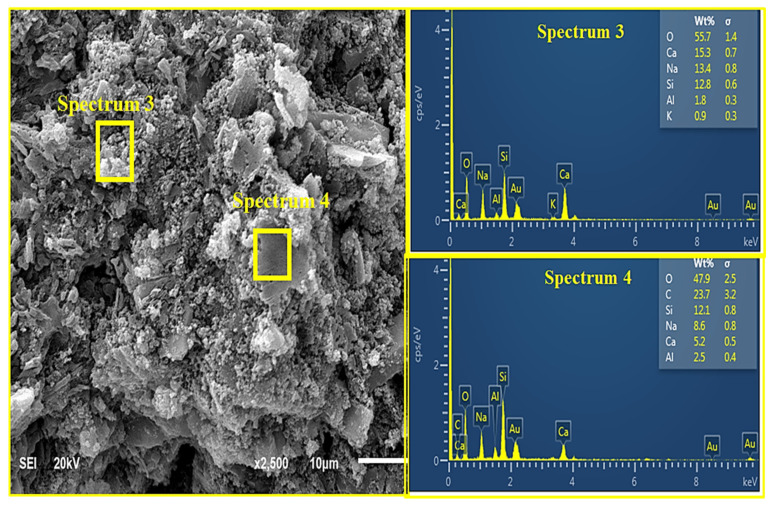
SEM + EDX result of AANL activated using combined NaSiO_3_ and 10 M NaOH.

Figure 9 and Figure 10 show the micrograph and EDX results of the AANLM binder, activated using only 10 M NaOH_(aq)_ (M_s_ = 0) and combined NaOH_(aq)_ and Na_2_SiO_3(aq)_ (M_s_ = 0.89) paste for the best and worst compressive strength results, respectively. Figure 9 shows the SEM and EDX pattern result of the binder using only 10M NaOH_(aq)_ (M_s_ = 0). The image reveals non-homogeneous and non-compacted structures with connected micropores. This indicates that the degree of polymerisation at low silica modulus (M_s_ = 0) is slow, resulting in the low compressive strength recorded in AANLM_0_. However, as the silica modulus increases, the SEM image for AANLM_1_ revealed a homogenous and denser microstructure due to more formation of alkaline activated products. The EDX results for AANLM_0_ indicates a higher ratio of Si/Na (7.89) at spectrum 1 compared to the values of Si/Na (0.96) at spectrum 3 and Si/Na (1.4) at spectrum 4 in AANLM_1_. This indicates the presence of excess unreacted silica gel in AANLM_0_. Furthermore, the Si/Ca (14.2) in AANLM_0_ (spectrum 1) is higher than the Si/Ca (2.33) present in AANLM_1_ (spectrum 4).

### 3.7. Effect of Curing Temperature on Strength and Microstructures of Alkali-Activated Volcanic Ash and Limestone Powder

#### 3.7.1. Effect of Curing Temperatures on Compressive Strength Development

**Figure 11:**
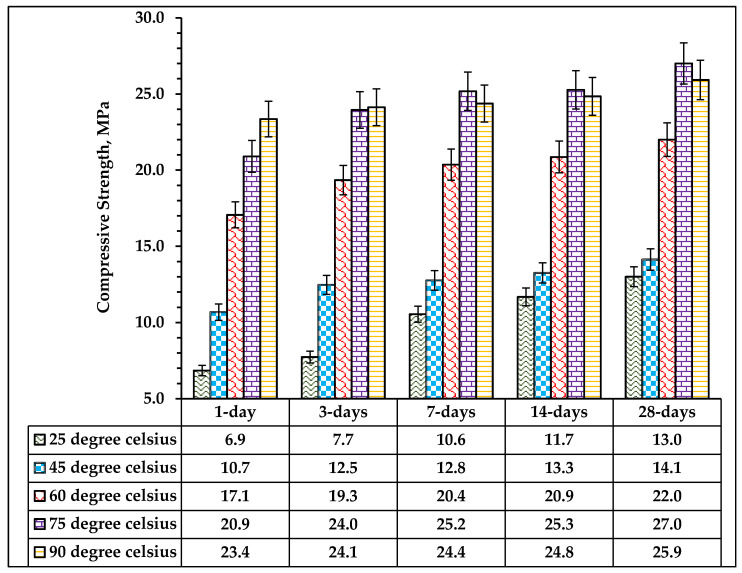
Effect of temperature on compressive strength of AANL mixes.

The compressive strength of alkali-activated limestone and volcanic ash powder mortar cured at various curing temperatures is revealed in Figure 11. Generally, the compressive strength for all the mixes increases with curing age as well as the curing temperature. It was observed that the early strength (one and three days) of the mortar increases with the corresponding increased in the curing temperature. The sample cured at room temperature (25 °C) exhibited the lowest compressive strength of 6.85 MPa and 7.73 MPa after 1 day and 3 days of curing. The 1-day compressive strength increased by 55.9%, 149.05%, 199.27% and 240.88% after curing at 45 °C, 60 °C, 75 °C and 90 °C, respectively. A similar trend was observed for the 3-days compressive strength. However, at 7, 14 and 28 days, the compressive strength increases at the temperature increased up to 75 °C, after which a dropped in the compressive strength was observed.

The maximum 28-days compressive strength of 27 MPa was achieved at the optimum curing temperature of 75 °C. Further increase in the curing temperature to 90 °C caused a reduction in the compressive strength by 4% due to drying effects. The curing temperature has a significant effect on the compressive strength development of alkali-activated mortar. An increase in temperature from room temperature of 25 °C to a higher curing temperature of 75 °C enhanced the geopolymerisation process of the alkaline activated binder.

#### 3.7.2. XRD Characterisation of Varied Silica Modulus on the Mortar Binder

**Figure 12:**
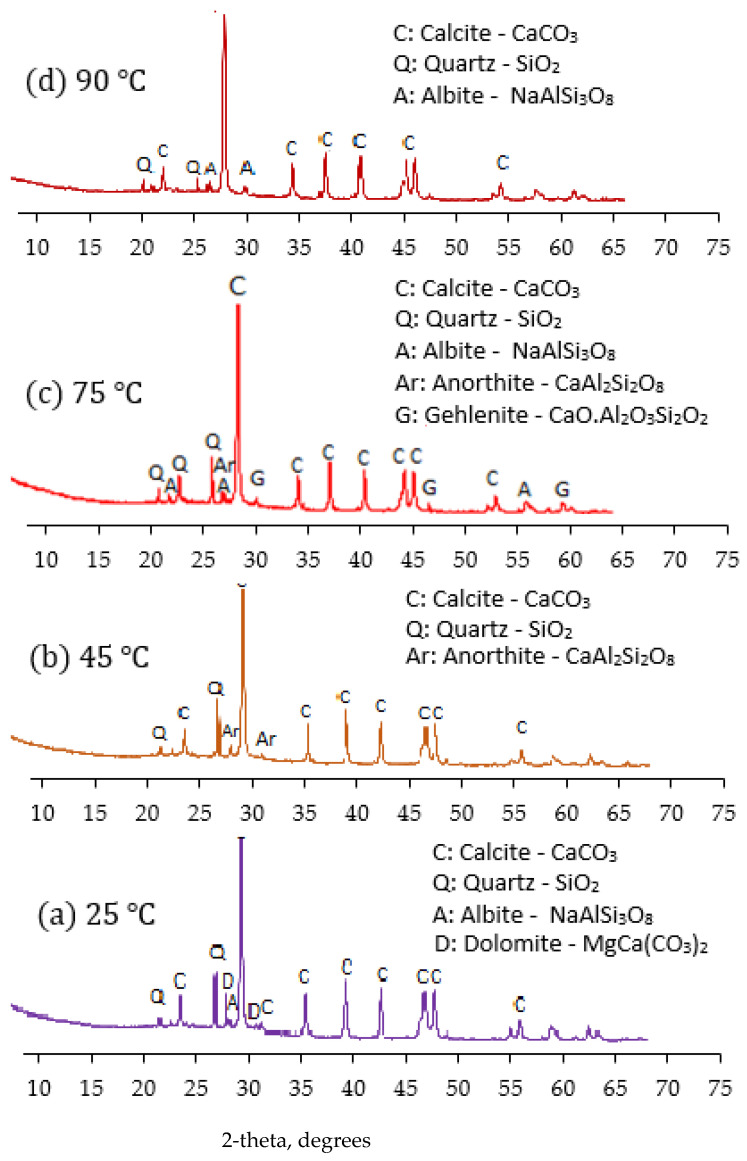
XRD of the activated product at different curing temperatures: (**a**) 25 °C; (**b**) 45 °C; (**c**) 75 °C; (**d**) 90 °C.

The XRD spectra of the binder cured at 25 °C, 45 °C, 75 °C and 90 °C are shown in Figure 12. The binder cured at room 25 °C lead to the formation of albite (NaAlSi_3_O_8_), calcite (CaCO_3_), dolomite (MgCa(CO_3_)_2_) and unreacted quartz (SiO_2_), as revealed in Figure 12a (25 °C). However, when the curing heat increased to 45 °C, anorthite (CaAl_2_Si_2_O_8_), calcite (CaCO_3_) and quartz (SiO_2_) were formed as revealed in Figure 12b. The anorthite present in this binder, which is similar to the C-A-S-H product, enhanced the slight strength development of the binder. Further increased in the curing temperature to 75 °C favoured the geopolymerisation process of the activated binder, which resulted in the formation of anorthite (CaAl_2_Si_2_O_8_), gehlenite (CaO.Al_2_O_3_.SiO_2_) and albite (NaAlSi_3_O_8_) as revealed in Figure 12c. The formation of anorthite (CaAl_2_Si_2_O_8_) and gehlenite (CaO.Al_2_O_3_.SiO_2_) compounds at the 2-theta angle of 27.35° and 32.34°, respectively, enhanced the strength development. However, upon an increase in the curing temperature to 90 °C, it was observed, as shown in Figure 12d, that the peak of quartz compound at 2- theta angle of 27.24° is lesser than the peak present in the sample cured at 75 °C. Furthermore, the anorthite (CaAl_2_Si_2_O_8_) and gehlenite (CaO.Al_2_O_3_.SiO_2_) compounds present in the binder 75 °C curing temperature disappeared at 90 °C curing temperature. This implies that at an optimum curing temperature of 75 °C, geopolymerisation and condensation of silica compounds present in the mixing matrix are better enhanced. This aforementioned process contributes positively to the high compressive recorded for the binder-activated binder cured at 75 °C. Therefore the optimum temperature for the synthesised binder is 75 °C.

#### 3.7.3. FTIR Analysis of Silica Modulus Effect

**Figure 13:**
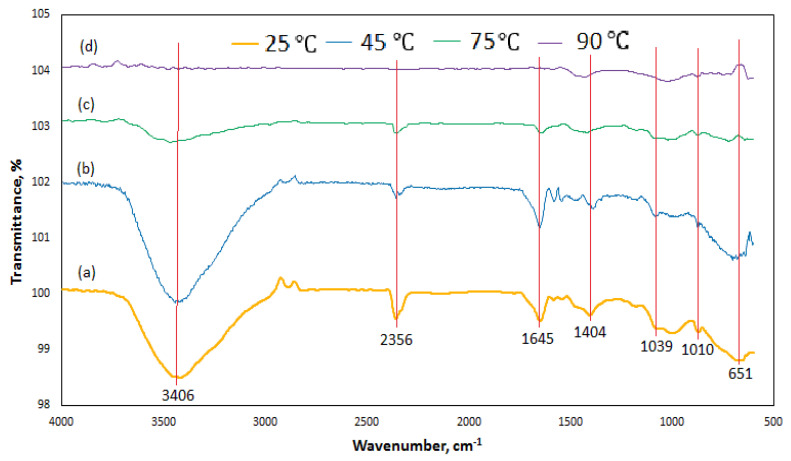
FTIR spectra of the activated product at different curing temperature (**a**) 25 °C; (**b**) 45 °C; (**c**) 75 °C; (**d**) 90 °C.

The FTIR spectra of the binder cured at 25 °C, 45 °C, 75 °C and 90 °C are shown in Figure 13. The spectra revealed some structural changes in the bond characteristics due to changes in curing temperature. From Figure 13a–c, the FTIR broad bands located at 3700–2900 cm^−1^ signify the presence of O–H stretching. The band is broader in both room cured samples and samples cured at 45 °C. The depth of the peak reduces as the curing temperature increases, and this peak disappeared at a curing temperature of 90 °C Figure 13d. Similar trends were observed for H–O–H bending that occurred between wavenumbers of 2380 and 2287 cm^−1^ and 1700 cm^−1^ to 1600 cm^−1^; this peak also disappeared at a curing temperature of 90 °C. The binder cured at room temperature and 45 °C had a broader trough compared to the one cured at 75 °C and 90 °C. This shows that there were more weak O–H and H–O–H bonds present in the former than the latter. This caused the low strength observed at lower curing temperatures.

Stretching vibration of C–O–O was observed between wavenumbers of 1420 and 1313 cm^−1^ in the samples cure at 25 °C, 45 °C, 75 °C and 90 °C. The depth of the peak reduces as the curing temperature increases. The FTIR pattern between 900 cm^−1^ and 1300 cm^−1^ associated with the Si–O–T (T = Si or Al) asymmetric vibration were also observed in the binder at all the curing temperature. In-plane bending vibration of C–O was also observed at a band of the weak and broad absorption peak of 657 cm^−1^.

#### 3.7.4. SEM + EDX Characterisation of Curing Temperature Effect

**Figure 14:**
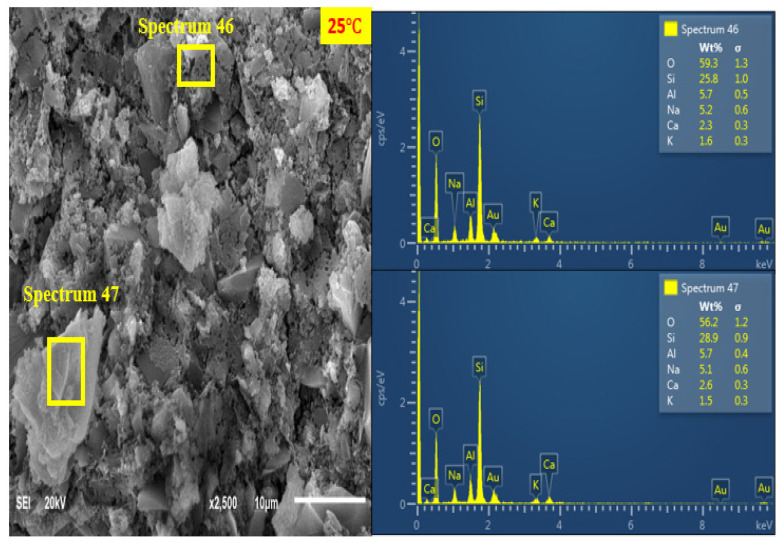
SEM + EDX result of AANL cured at 25 °C.

**Figure 15:**
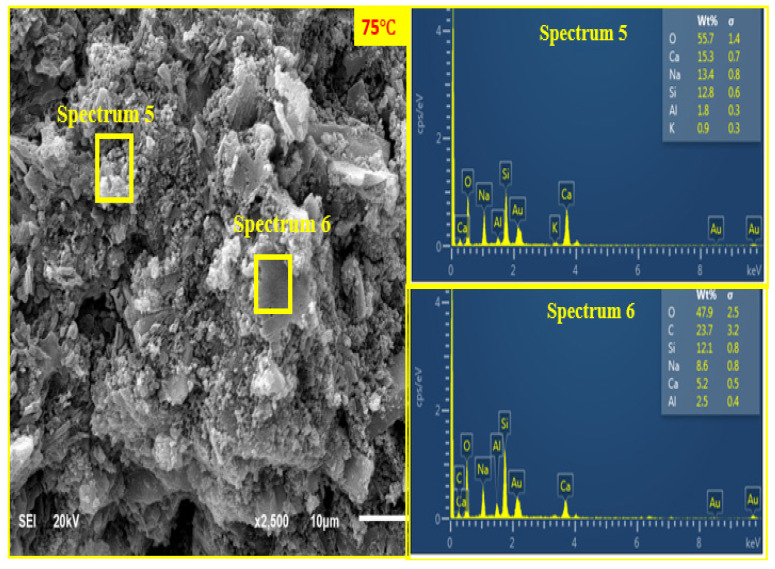
(SEM + EDX) of AANL cured at 75 °C.

**Figure 16:**
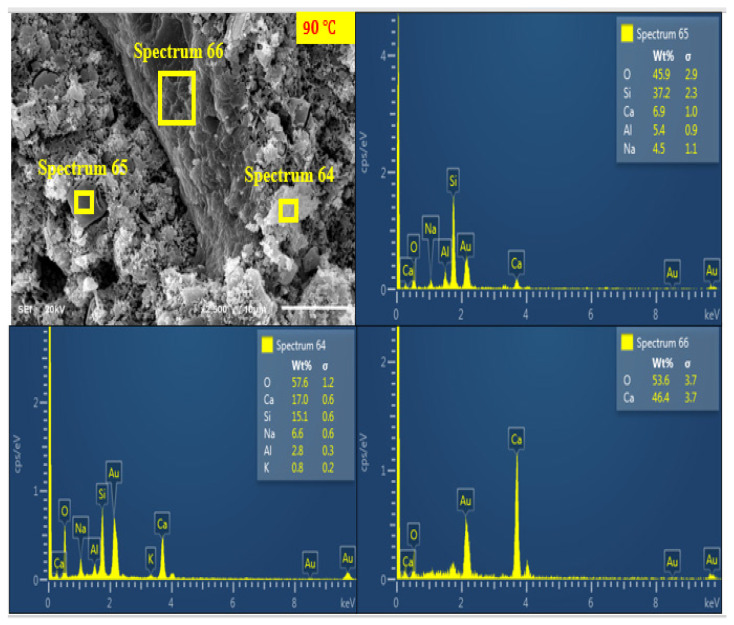
SEM + EDX of AANL cured at 90 °C.

Figure 14, Figure 15 and Figure 16 show the SEM and EDX analysis of AANL paste for the room cured sample, the optimum temperature (75 °C) and the sample cured above the optimum temperature (90 °C) respectively. Figure 14 shows the SEM and EDX result of the activated binder cured at 25 °C. The micrograph reveals a big size particle of non-homogeneous and non-compacted microstructure. This implies that the level of polymerization at low curing temperature such as room temperature does not favour the geopolymerisation process of alkaline activated LSP and VA, resulting in the low compressive strength recorded in the sample cured at room temperature. However, as the curing temperature increased to the optimum curing temperature of (75 °C), the SEM image (Figure 15) showed a homogenous and denser microstructure due to more formation of alkaline activated product. The EDX results for the sample cured at room temperature (25 °C) indicates a higher ratio of Si/Na (4.96) at spectrum 46 and Si/Na (5.67) at spectrum 47 compared to the values of Si/Na (0.96) at spectrum 5 and Si/Na (1.4) at spectrum 6 of sample cured at (75 °C). This indicates the presence of excess unreacted silica gel in AALN mortar cured at room temperature. Furthermore, the Si/Ca (12.54) in the room cured samples (spectrum 46) is higher than the Si/Ca (0.83) and 2.33 present in samples cured at the optimum temperature as revealed in spectrum 5 and spectrum 6, respectively. However, upon increasing the curing temperature to 90 °C, three distinct layers were observed in Figure 16, spectrum 64 revealed Si/Ca (0.89) slightly above the Si/Ca (0.83) obtained in sample cured at the optimum temperature, while spectrum 65 showed Si/Ca (5.4) higher than the one observed at the optimum curing temperature and lower than the one obtained at room cured temperature. This also implies that the formation of alkaline activated products was also formed at a 90 °C curing temperature; however, the formation of CaO at spectrum 66 showed the decomposition of the alkaline product could have occurred at 90 °C, which could have been responsible for the slight decline in the compressive strength observed at this particular curing temperature.

## 4. Conclusions

The impact of silica modulus (M_s_) and curing temperature on workability, compressive strength, bond properties, reaction products and the microstructures of alkali-activated binder synthesised from the binary blending of volcanic ash powder (VA) and limestone powder (LSP) were investigated. This study has contributed to understanding the impact of silica modulus and curing temperature on the strength and microstructural characteristics of the alkali-activated volcanic ash/limestone powder mortar. The utilisation of VA/LSP will be an addition to sustainable alternative binders and waste reduction.

The following conclusions were deduced:

1. The flow of the developed alkali-activated binder increases as silica modulus increased from zero to the optimum values of 0.89, and thereafter, a reduction in the workability was observed. All the flow values for all the developed binder were in an acceptable range of 135 mm to 220 mm;
2. The maximum strength of 27 MPa was obtained with silica modulus of 0.89 after 28 days of curing; however, the compressive strength reduced sharply above the optimum silica modulus due to the presence of more SiO_2_ than Na_2_O in the mix;
3. More than 70% of the 28-day compressive strength could be achieved within 12 h of curing with the usage of combined Na_2_SiO_3(aq)_/10 M NaOH at optimum M_s_;
4. Samples synthesised with sole 10 M NaOH_(aq)_ activator resulted in a binder with a low 28-day compressive strength (15 MPa) compared to combined usage of Na_2_SiO_3(aq)/_10 M NaOH_(aq)_ activators;
5. Usage of only sodium hydroxide solution as activator resulted in the formation of main calcite (CaCO_3_), quartz (SiO_2_), anorthite (Na_48_Ca_52_(Si_2.5_Al_1.5_)O_8_ and kaolinite (Al_2_Si_2_O_9_H_4_). However, usage of combined sodium hydroxide solution and sodium silicate as activator resulted in the formation of anorthite (CaAl_2_Si_2_O_8_), gehlenite (CaO.Al_2_O_3_.SiO_2_) and albite (NaAlSi_3_O_8_) that enhanced the strength development;
6. Usage of combined Na_2_SiO_3(aq)_/10 M NaOH_(aq)_ at an optimum (M_s_) of 0.81 enhanced microstructural densification of the product better than the usage of only NaOH_(aq)_;
7. Curing at low temperatures such as 25 °C and 45 °C hindered the geo-polymerisation process resulting in low 28-day compressive strength of 13 MPa and 14.13 MPa, respectively. In contrast, higher curing temperature positively enhanced strength development.

## Figures and Tables

**Table 1:** Chemical constituents of raw volcanic ash powder and limestone powder.

Oxides Components (%)	CaO	SiO_2_	Al_2_O_3_	Fe_2_O_3_	MgO	Na_2_O	K_2_O	L.O.I
LSP	94.1	2.5	0.8	1.2	0.6	-	0.3	44.0
VA	2.0	74.0	13.0	1.5	0.5	4.0	5.0	5.0

**Table 2:** Materials required in kg/m^3^ of alkali-activated volcanic ash/limestone powder mortar.

Mix #	Mix ID.	VA	LSP	NH Molarity	SS/NH	SS	NH	Water	Fine Aggregate
M1	AANLM_0_	242	363	10	0.0	0.0	303.0	60.5	1210
M2	AANLM_0.5_	242	363	10	0.5	101.0	202.0	60.5	1210
M3	AANLM_0.75_	242	363	10	0.75	129.9	173.1	60.5	1210
M4	AANLM_1.0_	242	363	10	1.0	151.5	151.5	60.5	1210
M5	AANLM_1.25_	242	363	10	1.25	168.3	134.7	60.5	1210
M6	AANLM_1.5_	242	363	10	1.5	181.8	121.2	60.5	1210

**Table 3:** Physical characteristics of raw volcanic ash powder and limestone powder.

Based Materials	LSP	VA
Specific gravity	2.70	2.30
Mean diameter (µm)	12.05	5.77
Specific surface area (cm^2^/g)	0.60	3.10
d90 (µm)	31.00	11.61
d50 (µm)	6.43	4.84
d10 (µm)	1.20	1.39

**Table 4:** Initial oxide composition of the alkaline activators.

Mix #	Mix ID.	Total SiO_2_, (kg/m^3^)	Total Na_2_O (kg/m^3^)	Total H_2_O (kg/m^3^)	SiO_2_/Na_2_O	H_2_O/SiO_2_	H_2_O/Na_2_O
M1	AANLM_0_	0.00	72.35	291.15	0.00	4.02	0.00
M2	AANLM_0.5_	29.42	57.08	277.00	0.52	4.85	9.41
M3	AANLM_0.75_	37.83	52.72	272.95	0.72	5.18	7.22
M4	AANLM_1.0_	44.13	49.45	269.92	0.89	5.46	6.12
M5	AANLM_1.25_	49.03	46.90	267.56	1.05	5.70	5.46
M6	AANLM_1.5_	52.96	44.87	265.68	1.18	5.92	5.02

## Data Availability

The raw data required to reproduce these findings are available in the cited references in Section 2.2 of this manuscript.
